# α-Synuclein and Anti-α-Synuclein Antibodies in Parkinson’s Disease, Atypical Parkinson Syndromes, REM Sleep Behavior Disorder, and Healthy Controls

**DOI:** 10.1371/journal.pone.0052285

**Published:** 2012-12-17

**Authors:** Lynnae M. Smith, Mya C. Schiess, Mary P. Coffey, Andrea C. Klaver, David A. Loeffler

**Affiliations:** 1 Department of Neurology Research, Beaumont Health System, Royal Oak, Michigan, United States of America; 2 Department of Neurology, University of Texas Houston Medical School, Houston, Texas, United States of America; 3 Department of Biostatistics, Beaumont Health System, Royal Oak, Michigan, United States of America; Massachusetts General Hospital/Harvard Medical School, United States of America

## Abstract

α-synuclein is thought to play a key role in Parkinson’s disease (PD) because it is the major protein in Lewy bodies, and because its gene mutations, duplication, and triplication are associated with early-onset PD. There are conflicting reports as to whether serum and plasma concentrations of α-synuclein and anti-α-synuclein antibodies differ between PD and control subjects. The objectives of this study were to compare the levels of α-synuclein and its antibodies between individuals with typical PD (n = 14), atypical Parkinson syndromes (n = 11), idiopathic rapid eye movement sleep behavior disorder (n = 10), and healthy controls (n = 9), to assess the strength of association between these serum proteins, and to determine group sizes needed for a high probability (80% power) of detecting statistical significance for 25% or 50% differences between typical PD and control subjects for these measurements. Analysis of log-transformed data found no statistically significant differences between groups for either α-synuclein or its antibodies. The concentrations of these proteins were weakly correlated (Spearman rho = 0.16). In subjects with typical PD and atypical Parkinson syndromes, anti-α-synuclein antibody levels above 1.5 µg/ml were detected only in subjects with no more than four years of clinical disease. Power analysis indicated that 236 and 73 samples per group would be required for an 80% probability that 25% and 50% differences, respectively, in mean α-synuclein levels between typical PD and control subjects would be statistically significant; for anti-α-synuclein antibodies, 283 and 87 samples per group would be required. Our findings are consistent with those previous studies which suggested that serum concentrations of α-synuclein and its antibodies are not significantly altered in PD.

## Introduction

α-synuclein is thought to play a prominent role in the pathogenesis of Parkinson’s disease (PD) because it is the major protein in Lewy bodies, the inclusions seen in ∼10% of pigmented (dopaminergic) neurons in the PD substantia nigra pars compacta [1], and because its gene mutations and multiplications are associated with early-onset, autosomal dominant PD [2]–[5]. α-synuclein expression is also increased in the brain in idiopathic PD [6]. This protein is synthesized by neurons in most regions of the brain and transported to presynaptic terminals, where it may play a role in neuronal plasticity, regulation of synaptic dopamine content, and/or neuroprotection [7], [8]. Soluble α-synuclein oligomers, rather than the fibrillar α-synuclein present in Lewy bodies, may be responsible for the early onset autosomal dominant form of PD [9].

Neurons secrete α-synuclein [10], resulting in its presence in CSF [11]. α-synuclein oligomer levels have been suggested as a possible biomarker for PD in CSF [12] and plasma [13]. α-synuclein is also detectable in peripheral blood, primarily in erythrocytes. Plasma contains <1% of blood α-synuclein [14], and the extent to which α-synuclein levels in plasma are derived from its CNS levels is unknown [15]. The literature contains conflicting reports as to whether total α-synuclein concentrations in serum and plasma differ between PD patients and healthy subjects [15]–[21], and widely varying levels of α-synuclein in peripheral blood have been reported, ranging from ∼78 pg/ml [16] to 250 ng/ml [17]. The status of antibodies to α-synuclein in PD subjects is also unclear, with some studies finding no change in these antibody levels between typical PD patients and controls [22], [23] while others have reported increased concentrations in PD patients [24]–[26]. The correlations (i.e., strengths of association) between the concentrations of α-synuclein and its antibodies in serum and plasma are unknown. Previous studies in which α-synuclein and anti-α-synuclein antibodies were compared between PD and control serum or plasma are summarized in Tables 1 and 2.

**Table 1:** PD serum and plasma α-synuclein measurements: previous studies.

Reference	Measurement	Method	Findings
El-Agnaf et al. [13]	Plasma α-synuclein oligomers	ELISA	Increased oligomeric α-synuclein in PD vs. controls
Shi et al. [15]	Plasma α-synuclein	Luminex bead assay	No differences between PD, AD, and controls
Lee et al. [16]	Plasma α-synuclein	ELISA (Amersham)	Increased levels in PD and MSA vs. controls; PD>MSA
Li et al. [17]	Plasma α-synuclein	Western Blot	Decreased levels in both early-onset and late-onset PD vs. controls
Duran et al. [18]	Plasma α-synuclein	ELISA (Invitrogen)	Increased levels in PD vs. controls; no effect of levodopa treatment on α-synuclein levels
Mollenhauer et al. [19]	Serum α-synuclein	ELISA	No differences between PD, MSA, DLB, PSP, neurological controls, and normal-pressure hydrocephalus
Foulds et al. [20]	Plasma α-synuclein	ELISA	No differences between PD and controls for total, oligomeric, or oligomeric phosphorylated α-synuclein; increased phosphorylated α-synuclein in PD vs. controls
Park et al. [21]	Plasma α-synuclein	ELISA	No differences between PD and neurologic controls for total or oligomeric α-synuclein

**Table 2:** PD serum anti-α-synuclein antibody measurements: previous studies.

Reference	Method	Findings
Woulfe et al. [22]	ELISA	No differences between PD and controls
Papachroni et al. [23]	Western blot	No differences in frequency between sporadic PD and controls; increased frequency of positive specimens in familial PD vs. controls
Gruden et al. [24]	ELISA	Increased in PD vs. controls, peaking at 5 yr
Yanamandra et al. [25]	ELISA, Western blot	Increased in early PD (Hoehn and Yahr 1–2) and late PD (Hoehn and Yahr 2.5–4) vs. controls; less increase in late PD
Gruden et al. [26]	ELISA	Increased in PD vs. control sera, associated with increased IL-6 and TNF-α and decreased IFN-γ

The present study was performed to address these issues. The objectives of the study were (1) to compare the concentrations of serum α-synuclein and anti-α-synuclein antibodies between subjects with typical PD, atypical Parkinson syndromes (APS), individuals with idiopathic rapid eye movement sleep behavior disorder (RBD) (which has been associated with an increased risk for developing PD [27], [28]), and healthy controls, (2) to measure the association between the serum levels of α-synuclein and its antibodies, and (3) to determine approximate group sizes that would have provided a high probability (80% power) to detect 25% or 50% mean differences between typical PD and control subjects for these measurements.

## Materials and Methods

### Study Subjects

All study subjects in this investigation provided written consent to participate under Institutional Review Board (IRB) - approved protocols (University of Texas Committee for the Protection of Human Subjects). All patients were assigned study numbers to assure de-identification of data. The serum samples were de-identified prior to their storage and subsequent shipment to Beaumont Hospital. The study was given exempt status by Beaumont Hospital’s Human Investigation Committee due to the lack of patient identifiers for the samples. Subjects were evaluated by MS, a board-certified neurologist and movement disorders specialist who directs the UT MOVE clinic and Movement Disorders fellowship program at the University of Texas - Houston. Serum samples were obtained from individuals with typical PD (n = 14), APS (n = 11), idiopathic RBD (n = 10), and clinically normal subjects (n = 9). The distinction between typical PD and APS was based upon the requisite clinical criteria for PD diagnosis [29]. The APS group included nine individuals with multiple system atrophy (MSA) ranging from mild to late stages of disease; five had a cerebellar subtype and the other four had a PD subtype. The others in the APS group were a subject with progressive supranuclear palsy (PS) and one with Lewy body dementia (DLB). Information obtained about each patient included age, gender, diagnosis, duration of clinical disease, and Parkinson’s disability scores (Unified Parkinson’s Disease Rating Scale (UPDRS) [30] total and motor scores, and the Hoehn and Yahr Scale [31] that quantifies parkinsonian features for all subjects). Demographic data for study subjects are summarized in Table 3. Blood samples were collected by vacutainer in red top tubes, allowed to clot for 30 min at room temperature, then centrifuged at 3000 rpm for 10 min. Serum samples were separated into 0.5 ml aliquots and stored at −80°C, then shipped on dry ice to the Neurology Research Laboratory of William Beaumont Hospital, where they were stored at −70°C.

**Table 3:** Subject demographic data.

	RBD	APS	PD	CTL
**N**	10	11	14	9
**Age (yr)**	58±9	59±10	63±9	55±8
**Age range (yr)**	(37–69)	(36–78)	(47–81)	(37–64)
**Gender (% males)**	70%	82%	71%	44%
**Duration (yr)**	NA	3 (2–15)	3.5 (1–12)	NA
**UPDRS total**	10.5 (3–22)	53 (40–71)^a^	25 (9–65)	1 (0–8)
**UPDRS motor**	2 (0–13)	30 (12–46)	17 (7–39)	0 (0–2)
**Hoehn and Yahr**	0 (0–1)	3.5 (2.5–5)^a^	1.3 (1–3.5)	0

Subject age, which was relatively normally distributed, is shown as mean±SD, together with age range. Gender is summarized as the percentage of male subjects in each group. Data for disease duration and Parkinson rating scales are presented as median and range because of the skewed nature of their distributions. Statistical comparisons relating to disease duration and Parkinson's disability scales were performed only between PD and APS patients. (RBD = rapid eye movement sleep behavior disorder; APS = atypical Parkinson syndromes; PD = typical Parkinson’s disease; CTL = clinically normal controls; UPDRS = Unified Parkinson’s Disease Rating Scale; NA = not applicable;

a p<0.05 vs. subjects with PD).

### Measurement of Serum α-synuclein

Serum α-synuclein concentrations were measured with the SensoLyte Anti-α-Synuclein (Human, Mouse, Rat) ELISA Kit (AnaSpec, Inc., Fremont, CA; cat. # 55550) following the manufacturer’s instructions. Sera were diluted 1∶20 and assayed in quadruplicate.

### Measurement of Serum Anti-α-synuclein Antibodies

Serum IgG concentrations to α-synuclein monomer were measured by enzyme - linked immunosorbent assay (ELISA) in 11 independent experiments. Each experiment included one sample from each of the four groups, except for the last two experiments which included variable sample numbers from each group depending on the number of samples remaining to be evaluated. α-synuclein monomer preparation and anti-α-synuclein antibody ELISAs were performed as reported previously [32]. α-synuclein monomer was resuspended to 1 µg/ml in Tris buffer (0.1 M, pH 8.8) and 100 µl/well was placed on a 96-well Nunc Maxisorp plate. As a “specificity control,” the same concentration of bovine serum albumin (BSA, Sigma-Aldrich) in Tris buffer was filtered and placed in adjacent wells. After incubation overnight at 4°C, serum samples, assayed in quadruplicate, were added to the plate. The samples were diluted 1∶10 in 0.01 M phosphate buffer (PBS), pH 7.2, with 0.1% Tween-20 and 1% BSA. Serum from a clinically normal individual was included in each experiment to permit normalization of data between experiments. Four-fold dilutions of rabbit anti-α-synuclein antibody (Millipore, Billerica, MA), from 1∶1,000 (2,000 ng/ml) to 1∶256,000 (7.8 ng/ml) were included as the standard curve on each plate. Secondary antisera were biotinylated goat anti-human IgG (cat. # 109-065-098, Fc(gamma) fragment specific; Jackson Immunoresearch Laboratories, West Grove, PA; 1∶1,000 dilution) for wells with human serum samples and biotinylated goat anti-rabbit IgG (Vector, 1∶1,000) for wells with rabbit anti-α-synuclein antibody. After incubation with streptavidin-alkaline phosphatase (Zymed Laboratories, Invitrogen, Carlsbad, CA; 1∶1,000 in PBS-T), para-nitrophenol phosphate (Sigma) was added (5 mg in 40 ml of 1 M diethanolamine buffer, pH 9.8) and the plate was read at 405 nm with a Vmax kinetic microplate reader (Molecular Devices Corp., Sunnyvale, CA) until the standard curve OD reached 1.0±0.1. To calculate specific anti-α-synuclein monomer antibody concentrations, the mean antibody concentration measured when each serum sample was incubated on BSA-coated wells was subtracted from antibody concentrations measured on wells coated with α-synuclein monomer.

### Statistical Procedures

Between-group differences for α-synuclein and anti-α-synuclein antibody concentrations were evaluated for statistical significance by one-way analysis of variance (ANOVA) on log-transformed data, because the assumptions for the ANOVA (normal distribution and equal variances) were more closely met on log-scale data than on the raw data. Differences in demographic variables (except for gender distribution) in the study groups were compared with the Kruskal-Wallis test or Wilcoxon Rank Sum test; for disease duration and Parkinson’s disability rating scales, only PD and APS patients were compared. Associations between variables were examined with Spearman’s rank-order correlation coefficient rho, a nonparametric measure, due to skewness in the antibody and α-synuclein protein data. Significance was set at p<0.05 for all analyses. All reported p-values are two-sided. Receiver operating characteristic (ROC) curves were generated with SAS using the LOGISTIC procedure [33] to assess the usefulness of our measurements of serum α-synuclein or anti-α-synuclein antibody concentrations as biomarkers for clinical diagnosis of PD; the data from typical PD and healthy control subjects were used to fit these curves.

### Power and Sample Size Analysis

Calculations were based on a significance level of 0.05, with 80% power to detect specified differences (25% or 50%) in mean concentrations of α-synuclein or specific anti-α-synuclein antibodies between typical PD patients and healthy controls. The analysis assumed that inference would be done on the log scale data; in this analysis, the ratio of the treatment mean (i.e., PD mean) to the control mean that one wishes to detect is specified, as well as the coefficient of variation (CV). CVs were calculated for the α-synuclein protein and anti-α-synuclein antibody concentrations for the four groups in this study, and for both sample size analyses, the largest CV among the groups was used. (The CVs used were 1.05 for the α-synuclein analysis and 1.20 for the antibody analysis.) The calculations used the sample size procedures in NCSS PASS 11 software [34] for “Inequality Tests for Two Means using Ratios (Two-Sample T-Test).”

## Results

### Patient Demographics

The average age of the study subjects (Table 3) was 59.4 years old (standard deviation = 9.3 years, median = 59, range = 36–81 years). The age differences between groups were not statistically significant (Kruskal-Wallis test: p = 0.16). 68% of study participants were male. In the combined group of 14 typical PD and 11 APS subjects, 18 patients (72%) had clinical symptoms for ≤5 years, five (20%) had symptoms for 6–10 years, and two (8%) had symptoms for more than 10 years. Parkinson’s disability ratings tended to be higher in APS than in typical PD patients (UPDRS total scores: p = 0.04; UPDRS motor scores: p = 0.055; Hoehn and Yahr scores: p = 0.002 [Wilcoxon Rank Sum test]).

### Serum α-synuclein

**Figure 1:**
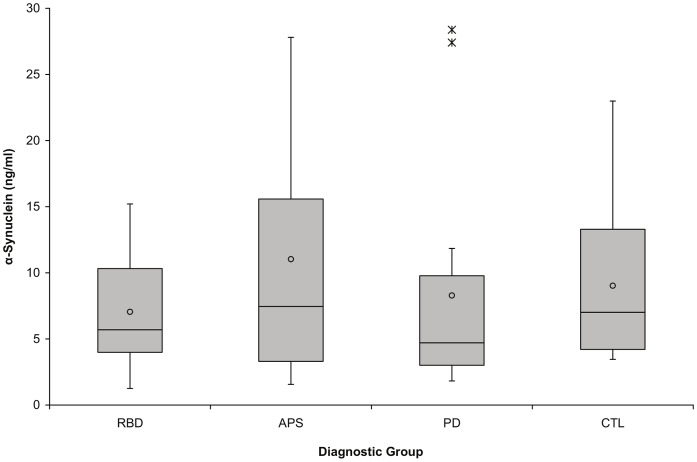
Serum α-synuclein levels do not significantly differ between subjects with rapid eye movement sleep behavior disorder, atypical Parkinson syndromes, typical Parkinson’s disease, and normal controls. Group means (circle), medians (line through center of box), upper and lower quartiles (upper and lower borders of box, respectively), most extreme non-outlier values (lines extending from box), and outliers (asterisks) are shown for α-synuclein measurements for each group. There were no significant differences between groups when log-transformed data were examined (p = 0.70). (RBD = rapid eye movement sleep behavior disorder; APS = atypical Parkinson syndromes; PD = typical Parkinson’s disease; CTL = clinically normal controls).

A box plot of the serum α-synuclein results (group means, medians, upper and lower quartiles, most extreme non-outlier values, and outliers) is shown in Fig. 1. There were no significant differences between groups when log-transformed data were examined (p = 0.70). No relationship was found between serum α-synuclein and subject age (all subjects), disease duration in typical PD and APS subjects, or disability ratings in typical PD and APS subjects. Spearman’s rank - order correlation coefficients for α-synuclein concentrations with these variables were: subject age, −0.06; disease duration, 0.23; UPDRS total score, 0.20; UPDRS motor score, 0.24; and Hoehn and Yahr score, 0.21 (all p>0.25; no patterns seen in scatter plots). Serum α-synuclein levels were similar between males and females (p = 0.24; median α-synuclein values: males, 6.39 ng/ml, females, 4.48 ng/ml).

Two PD subjects had elevated serum α-synuclein levels that were statistical outliers relative to the PD group on the original scale but not on the log-transformed scale which was used for statistical analysis. One of these individuals was a 65-year-old female with 4-year duration of tremor-dominant PD who exhibited depression and anxiety; her disability rating scores were UPDRS total = 53, UPDRS motor = 34, and Hoehn and Yahr = 3.5. The other subject was a 47-year-old male with 8-year PD duration (also tremor-dominant) and scores of UPDRS total = 62, UPDRS motor = 17, and Hoehn and Yahr = 1.

### Serum Anti- α -synuclein Antibodies

**Figure 2:**
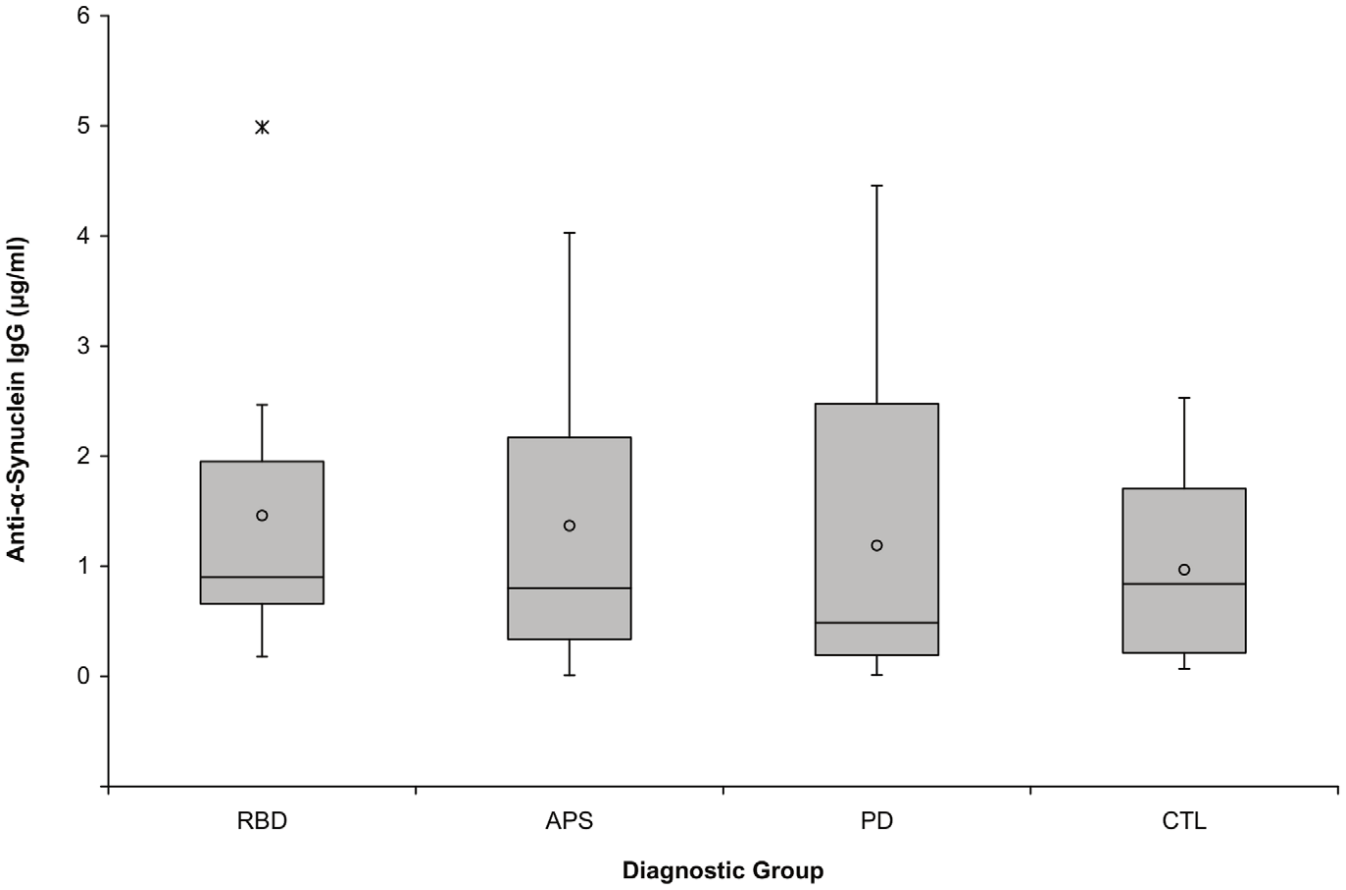
Serum anti-α-synuclein IgG levels do not significantly differ between subjects with rapid eye movement sleep behavior disorder, atypical Parkinson syndromes, typical Parkinson’s disease, and normal controls. Designations for group means, medians, upper and lower quartiles, most extreme non-outlier values, and outliers are as for Fig. 1. There were no statistically significant differences between group means for these parameters when log-transformed data were examined (p = 0.69). (RBD = rapid eye movement sleep behavior disorder; APS = atypical Parkinson syndromes; PD = typical Parkinson’s disease; CTL = clinically normal controls).

Results for specific antibody binding to α-synuclein are shown in Fig. 2. There were no significant differences between groups for log-transformed data (p = 0.69). Group mean values for nonspecific binding of serum samples (i.e., to BSA-coated wells) ranged from 28% to 41% of their total serum binding to α-synuclein (data not shown). No relationship was evident between specific anti-α-synuclein antibody levels and subject age (pooled data from all subjects) or disability ratings (typical PD and subjects with APS only). Spearman’s rho values for the association of anti-α-synuclein antibodies with these variables were: age = 0.01, UPDRS total = 0.03, UPDRS motor = 0.06, and Hoehn and Yahr scores = 0.06 (all p>0.75). The Spearman’s rho value for specific antibody binding and disease duration was −0.19 (p = 0.35). Within the RBD group, the anti-α-synuclein antibody concentration in a 65-year-old female was sufficiently elevated to be a statistical outlier on the original scale but not on the log scale used for analysis. This individual had developed RBD within the past five years; she was not symptomatic for PD, PSP, MSA, or cognitive decline. Her UPDRS motor score of 5 was due to an action tremor and mild tremulousness.

**Figure 3:**
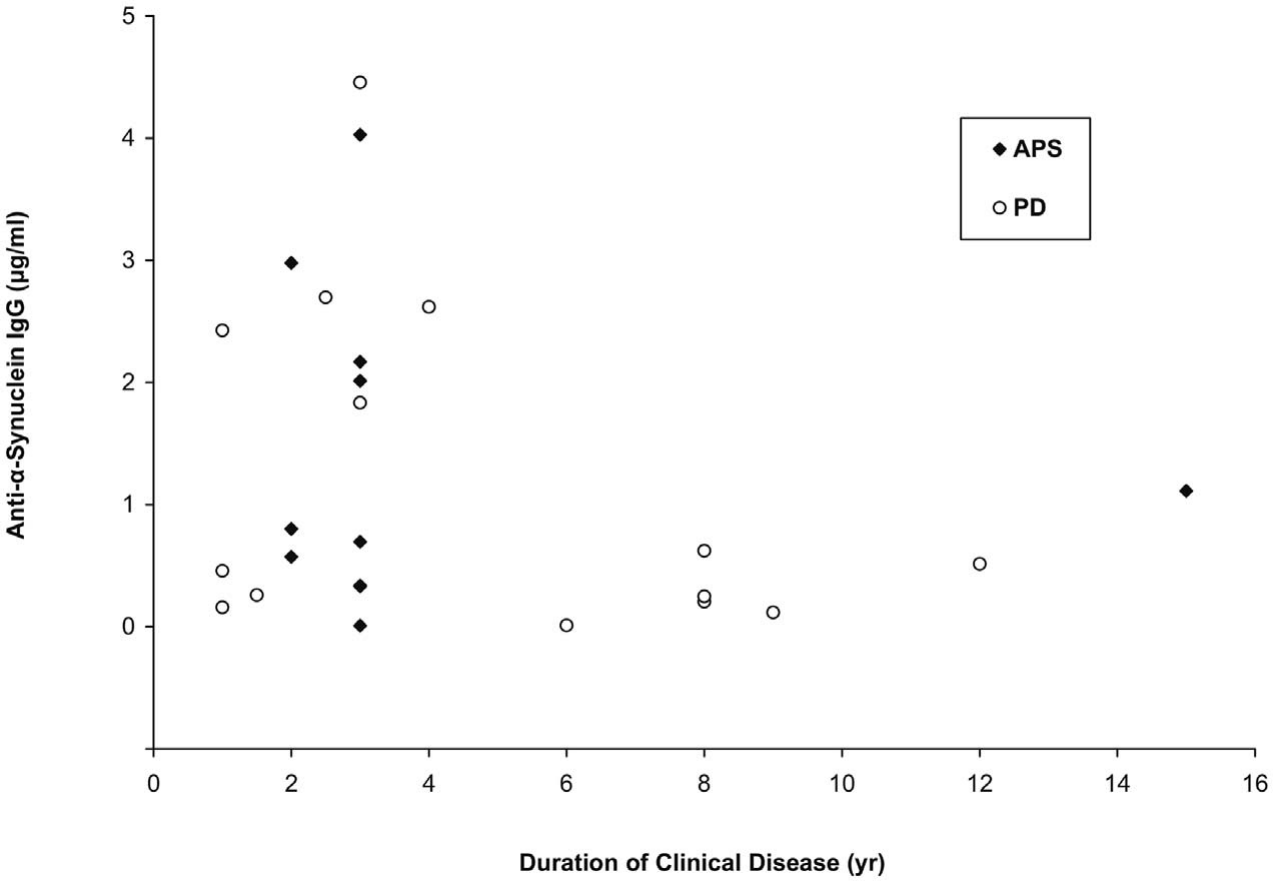
Anti-α-synuclein IgG concentrations in subjects with typical Parkinson’s disease and atypical Parkinson syndromes appear to decline with increased duration of clinical disease. Antibody concentrations above 1.5 µg/ml were only found in subjects with disease duration ≤ four years. (Diamonds = atypical Parkinson syndromes [APS]; circles = typical Parkinson’s disease [PD]).

Examination of scatter plots of total and specific antibody levels versus disease duration for typical PD and APS patients indicated that the highest antibody levels were present in patients with no more than four years of clinical disease. The scatter plot for specific antibody concentrations vs. disease duration is shown in Fig. 3. As was the case for α-synuclein, the distribution of anti-α-synuclein antibodies was similar between male and female subjects (p = 0.30; median α-synuclein values: males, 0.73 µg/ml, females, 0.92 µg/ml).

### Correlation between Serum α-synuclein and Anti-α-synuclein Antibody Concentrations

The concentrations of serum α-synuclein and its specific antibodies were weakly associated (Spearman’s rho = 0.16; p = 0.29). Scatterplots (not shown) did not suggest any relationships between these variables in pooled data or in individual groups.

### ROC Curves

**Figure 4:**
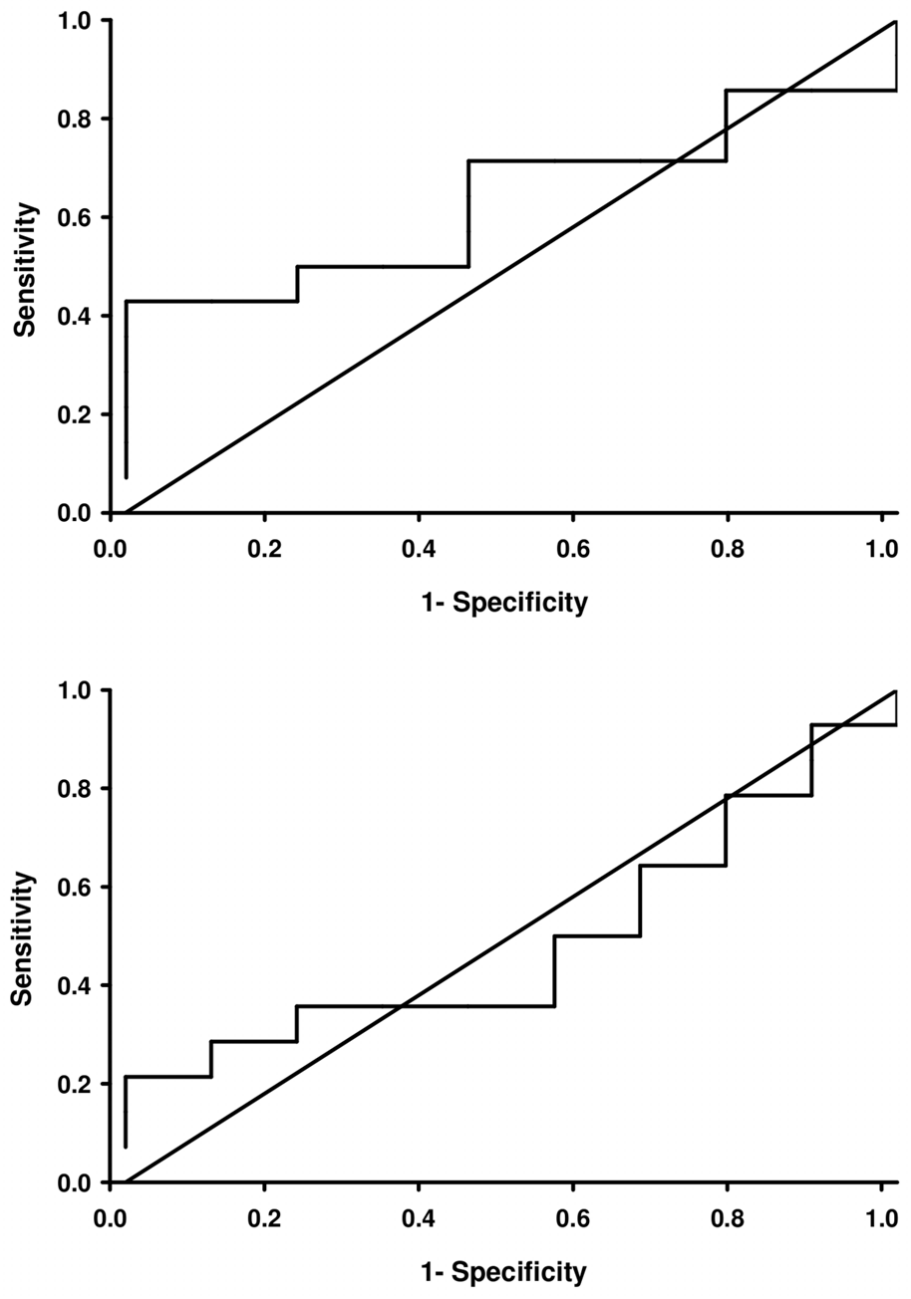
Receiver operating characteristic (ROC) curves provide no evidence that measurements of serum α-synuclein or anti-α-synuclein antibody concentrations can discriminate between individuals with typical Parkinson’s disease and normal control subjects. Curves for α-synuclein and anti-α-synuclein antibody concentrations are shown in Figs. 4a (top) and 4b (bottom). The areas under the curve (AUC) for the two curves were 0.64 and 0.49, respectively.

ROC curves for α-synuclein and anti-α-synuclein antibody concentrations are shown in Fig. 4. The areas under the curve (AUC) were 0.64 and 0.49, respectively, for the two proteins. Neither measure provided acceptable discrimination between typical PD patients and healthy control subjects.

### Power and Sample Size Analysis

Power analyses indicated that for serum α-synuclein, a two-sided t test on log -transformed data at the p = 0.05 level with 236 and 73 samples per group would achieve 80% power to detect 25% and 50% increases, respectively, in mean values between typical PD and healthy control subjects with a CV on the original scale of 1.05. For anti-α-synuclein antibodies, 283 and 87 samples per group would achieve 80% power to detect 25% and 50% increases in mean values between the two groups with a CV on the original scale of 1.20.

## Discussion

This study found no evidence for differences in the concentrations of serum α-synuclein or anti-α-synuclein antibodies between typical and atypical PD patients, individuals with RBD, and healthy controls, and no evidence of a relationship was noted between these protein levels and PD disability ratings. The lack of alterations in α-synuclein levels between PD patients and controls in this study agrees with earlier reports by Shi et al. [15], Mollenhauer et al. [19], Foulds et al. [20] and Park et al. [21], but disagrees with studies by Lee et al. [16] and Duran et al. [18], both of whom found increased α-synuclein concentrations in PD, and Li et al. [17], who found decreased levels. Serum α-synuclein concentrations in the present study ranged from 1.3–28.4 ng/ml, similar to those reported by Duran et al. [18] (12–17 ng/ml), Mollenhauer et al. [19] (10.3–12.6 ng/ml), and Laske et al. [35] (4.7–8.1 ng/ml; measured only in DLB, AD, and controls). The α-synuclein levels in the latter three studies and the present one were far greater than those reported by Lee et al. [16] (76–80 pg/ml) and lower than those reported by Shi et al. [15] (37–49 ng/ml) and Li et al. [17] (26–250 ng/ml).

The two elevated values for serum α-synuclein that were statistical outliers on the original scale were found in PD patients; however, these values were not outliers on the log-transformed scale used for analysis. Although both of these individuals had higher total UPDRS scores than the PD median scores, the connection between their α-synuclein levels and their increased PD-related disability is unknown because our analysis found no evidence for a relationship between serum α-synuclein and PD disability ratings in study subjects with parkinsonian features. One of the outlier values was from a 47-year-old male, who was the youngest patient in the PD group and the only one to have early-onset parkinsonism, defined as parkinsonism starting before the age of 40 [36].

We also found no significant differences between groups for anti-α-synuclein antibody levels. The lone statistical outlier value (on the original scale only, but not the log scale) among the anti-α-synuclein measurements was from a patient with RBD, but there was nothing in her clinical history that suggested a reason for her elevated antibody level. The lack of change in anti-α-synuclein antibodies in our PD patients agrees with earlier reports by Woulfe et al. [22] and Papachroni et al. [23] but conflicts with the findings of Gruden, Kanamandra, and colleagues [24]–[26] who reported increased anti-α-synuclein antibody levels in PD patients relative to healthy controls. (In the study by Papachroni et al. [23], antibody detection was by western blot; no differences were found for the incidence of individuals with anti-α-synuclein antibodies between idiopathic PD and controls, although this incidence was increased in subjects with familial PD.) Gruden et al. [24] found that these antibodies, while increased in five-year PD patients, returned to control levels in ten-year PD patients, while Yanamandra et al. [25] reported that the magnitude of the increase in anti-α-synuclein antibody levels in PD patients tended to be less in “late PD” patients (Hoehn and Yahr scores 2.5–4; mean increase vs. controls of 6-fold for mean antibody levels to α-synuclein monomer) than in “early PD” patients (Hoehn and Yahr scores 1–2; mean increase vs. controls of 8-fold for anti-α-synuclein monomer antibody levels). More recently, Gruden et al. [26] reported that increased anti-α-synuclein antibodies in PD sera were associated with elevations in interleukin-6 (IL-6) and tumor necrosis factor-α (TNF-α) concentrations and decreased interferon-γ (IFN-γ) levels. We observed a trend similar to that reported in the initial paper by Gruden et al. [24] in that the highest anti-α-synuclein antibody levels in our study among subjects with parkinsonian features were in patients with clinical disease duration of ≤ four years, although the levels of these antibodies in approximately half of these “short duration” patients were comparable to the antibody levels in PD patients (and in one APS patient) with disease duration>four years. The mechanism underlying this trend for reduction of anti-α-synuclein antibodies with increased PD duration is unknown. The initial investigation by Gruden et al. [24] also reported decreased numbers of T and B lymphocytes in PD patients and suggested that this might contribute to their finding that antibodies to α-synuclein returned to control levels in ten-year PD patients; however, they found no differences in T or B lymphocyte numbers between five-year and ten-year PD patients. The significance of possible reductions in anti-α-synuclein antibody levels in PD patients as their disease progresses will depend upon the neuroprotective effects of these antibodies. We recently reported [37] that intravenous immunoglobulin (IVIG), which is composed of pooled immunoglobulins (primarily IgG) from thousands of healthy donors and contains antibodies to α-synuclein monomer and soluble oligomers [32], significantly reduced the *in vitro* neurotoxicity of α-synuclein oligomers to human neuroblastoma cells, although it did not prevent α-synuclein aggregation. The extent to which anti-α-synuclein antibody levels in serum reflect the levels of these antibodies in the CNS, and the possible neuroprotective effects of these antibodies against α-synclein’s neurotoxic effects in the brain, are unknown.

In contrast to findings by Yanamandra et al. [25], we found no relationship between anti-α-synuclein antibodies and disability ratings for our PD and APS patients. The conflicting results between these studies could be due in part to small sample sizes and/or methodological differences, for example in the preparation of α-synuclein which was used to detect antibody binding. Apparently none of the earlier ELISA studies subtracted nonspecific antibody binding, which we found to account for 28–41% of total serum binding to α-synuclein among the various groups. The mean concentrations for specific anti-α-synuclein antibodies for the groups in this study ranged from 0.97 to 1.46 µg/ml, higher than reported by Woulfe et al. [22] (PD = 0.178 µg/ml, controls = 0.216 µg/ml). To our knowledge, the latter study is the only previous one in which anti-α-synuclein antibody concentrations, as opposed to percent of positive samples in each group [23] or titers [24]–[26], have been reported. (Maetzler et al. [38], measuring serum anti-α-synuclein antibodies in subjects with PD and dementia, DLB, Alzheimer’s disease [AD] and frontotemporal dementia [FTD], vascular dementia, and healthy controls, reported results as log α-synuclein IgG/total IgG, so comparison of these findings to our results and to those of Woulfe et al. [22] is difficult.).

Serum α-synuclein and anti-α-synuclein antibody concentrations were weakly correlated in this study. There is no reason why these protein levels should necessarily be strongly correlated; increased antibody levels to α-synuclein could promote its clearance, thus lowering its serum concentration. In addition, some of the anti-α-synuclein antibodies measured in this study may actually have been generated against other antigens; for example, antibodies generated against a membrane protein of Epstein-Barr virus have been reported to crossreact with α-synuclein and to stain Lewy bodies [39]. Alternatively, because our α-synuclein ELISA detected total soluble α-synuclein, the various α-synuclein conformations in peripheral blood might differ in terms of their abilities to stimulate an antibody response. The conformation of endogenous human α-synuclein in physiological conditions is unclear; Bartels et al. [40] recently suggested that it may exist primarily as a tetramer, but Fauvet et al. [41] has claimed that human α-synuclein of neuronal origin exists primarily as a monomer. Although α-synuclein monomer was used to coat the wells in our antibody ELISA, the extent to which antibodies to other soluble α-synuclein conformations may also have been detected is unknown.

To our knowledge, power analyses of appropriate group sizes for comparing serum α-synuclein and its antibodies between PD patients and controls have not been previously published. Our power analyses indicated that very large sample sizes (>230 samples/group) would have been required for 25% differences in group means to have an 80% probability of being statistically significant. Negative findings in some of the previous studies and in the current study could be due to low statistical power, given how small the actual sample sizes were relative to the sample sizes needed for 80% power. Because of our small sample sizes, the failure to achieve statistical significance in this study was not particularly informative; however, the observed differences were quite small. In addition, the possibility remains that, if larger sample sizes had been analyzed, subpopulations within the typical PD, APS, or RBD groups might have been found whose mean values for the proteins differed from control mean values. Our sample sizes were too small to have a reasonable chance to detect such a possibility.

In conclusion, this study found no evidence for differences in serum α-synuclein and anti-α-synuclein antibodies between subjects with typical PD, atypical Parkinson’s syndromes, RBD, and healthy controls. Further, our data suggest that the levels of these serum proteins are only weakly associated. The discrepancies for the levels of serum and plasma α-synuclein reported in the literature suggest that standardized methods should be adopted for these measurements; they also suggest the need for a well-designed study with large enough sample sizes to have a reasonable probability of detecting differences of clinical interest. The possible neuroprotective effects of anti-α-synuclein antibodies must be better understood to determine whether therapeutic approaches to increase these antibodies in PD and in other synucleinopathies [42], [43] should be further explored.
